# Chinese English teachers’ occupational intention during distance education: The role of burnout and job-related stress

**DOI:** 10.3389/fpsyg.2022.1024250

**Published:** 2022-10-06

**Authors:** Bo Zhang

**Affiliations:** College of Foreign Languages, Zhoukou Normal University, Zhoukou, China

**Keywords:** job-related stress, English teachers, distance education, China, turnover intention, burnout

## Abstract

Since turnover intention has a negative impact on teachers’ productivity, a bulk of educational research has studied the personal, organizational, and emotional predictors of this construct. Nevertheless, the predictive function of burnout and job-related stress as two emotional factors has been less attended to by scholars. To address this gap, the current empirical study explored the role of burnout and job-related stress in Chinese English teachers’ turnover intention during distance education. In doing so, three pre-designed questionnaires were distributed among 221 Chinese teachers. Having collected the needed data, the researcher analyzed the datasets through the Pearson correlation test and multiple regression analysis. As a result of the correlation test, positive, direct relationships were found between burnout, job-related stress, and teacher turnover intention. Moreover, the results of the regression analysis evinced the significant role of burnout and job-related stress in predicting Chinese English teachers’ turnover intention. The implications of the results are thoroughly discussed.

## Introduction

With the COVID-19 outbreak, educational institutions throughout the world were forced to choose distance education with huge changes in the learning and teaching methods (Derakhshan, 2021; Moser et al., 2021). Likewise, educational institutions in China, including schools, colleges, and universities, were also forced to undergo an immediate transition to remote educational settings in order to mitigate the spread of the Coronavirus among stakeholders (Gao and Zhang, 2020; Hong et al., 2021). The abrupt transition from face-to-face to remote education environments confronted educational institutions with a range of serious challenges (Shahnama et al., 2021; Silva et al., 2021), including increased turnover intention among teachers (Collie, 2022; Matthews et al., 2022). Teachers’ turnover intention, also known as teachers’ intention to leave, generally pertains to teachers’ reluctance to pursue the teaching career (Liu and Onwuegbuzie, 2012). For McInerney et al. (2015), teachers’ turnover intention refers to “teachers’ intention to leave the educational institution they are working for or to quit the teaching profession and move to a different career pathway” (p. 11). Teachers’ turnover intention is of high importance to educational managers due to the detrimental impact it may have on teachers’ productivity, students’ learning outcomes, and the educational institution itself (Ronfeldt et al., 2013; Adnot et al., 2017). Owing to the negative effects of teachers’ turnover intention on teaching quality and student achievement, identifying and managing the predictors of this construct seems crucial.

As put by Liu and Onwuegbuzie (2012), teachers’ turnover intention is subject to different organizational, personal, and emotional factors. Considering this point, many educational scholars have studied various organizational and personal factors as predictors of teachers’ turnover intention (e.g., Klassen and Chiu, 2011; You and Conley, 2015; Arnup and Bowles, 2016; De Neve and Devos, 2017; Wang and Hu, 2017; Rothmann and Fouché, 2018; Zhou et al., 2020; Nazari and Alizadeh, 2021, to cite a few). Similarly, several researchers have evaluated the emotional predictors of this construct (e.g., Larkin, 2015; Shah and Jumani, 2015; Imran et al., 2017; Park and Johnson, 2019; Kartika and Purba, 2022, among others). Yet, the predicting role of burnout and job-related stress as two emotional factors has been less attended to by researchers. To respond to this gap, the current investigation seeks to scrutinize the role of burnout and job-related stress in English teachers’ turnover intention.

As a probable predictor of turnover intention, burnout generally pertains to a state of emotional, physical, and psychological exhaustion resulting from prolonged engagement in emotionally demanding and stressful activities (Jennett et al., 2003; Schaufeli et al., 2009). With respect to this definition, Kyriacou (2015) characterized teacher burnout as “a syndrome of physical, emotional, and attitudinal exhaustion toward the teaching profession, which results from experiencing stress over a long period” (p. 72). As put forward by Shoji et al. (2016), teachers’ burnout may negatively affect their self-efficacy beliefs in that it gradually causes teachers to feel as if they do not have the sufficient personal resources to improve their students’ learning outcomes. Teachers’ burnout, according to Wong et al. (2017), may also hinder their productivity as it makes them indifferent to students and their academic achievements. Additionally, as noted by Lu and Gursoy (2016), burnout discourages teachers from continuing their vocation by leading them toward physical and emotional exhaustion.

Closely related to burnout, job-related stress is another potential determinant of turnover intention that pertains to “the level of pressure and demands made upon an employee in the working environment” (Jepson and Forrest, 2006, p. 185). In the teaching profession, job-related stress deals with teachers’ unpleasant emotional states caused by work overload or professional responsibilities (Kyriacou, 2011). Job-related stress is believed to be closely related to teachers’ emotional exhaustion, job dissatisfaction, work disengagement, and attrition (Barouch Gilbert et al., 2014; Reilly et al., 2014; Yu et al., 2015). Furthermore, as pointed out by Skaalvik and Skaalvik (2016), job-related stress has also something to do with teachers’ intention to leave the profession. They argued that feeling too much stress in the workplace substantially increases teachers’ willingness to quit the teaching profession.

Due to the significant role that burnout and job-related stress play in decreasing teachers’ turnover intention (Lu and Gursoy, 2016; Skaalvik and Skaalvik, 2016), some educational scholars have empirically studied the function of these two emotional constructs in teachers’ turnover intention (Chika et al., 2016; Xu et al., 2017; Jang and Kim, 2019; Lee, 2019; Mingming et al., 2021; Lai et al., 2022). However, few scholars have pursued this line of research in the field of language education. Simply said, there is a dearth of research evaluating the function of burnout and job-related stress in language teachers’ turnover intention. Moreover, as the review of previous studies revealed, no inquiry has examined the role of these job-related variables in language teachers’ turnover intention in the context of distance education. Therefore, the current research aims to address these lacunas by scrutinizing the potential role of burnout and job-related stress in Chinese English teachers’ turnover intention during distance education. To accomplish this, two research questions were formulated:

RQ1. Is there any significant association between burnout, job-related stress, and Chinese teachers’ turnover intention?

RQ2. To what extent do burnout and job-related stress significantly predict Chinese English teachers’ turnover intention during distance education?

## Literature review

### Theoretical model

The predictive role of job-related stress and burnout in teachers’ turnover intention appears to be justified through the “Job Demands-Resources (JD-R)” model (Bakker and Demerouti, 2007). This model is generally grounded on the idea that each profession has some distinguishing features that can be classified as job resources or job demands (Demerouti et al., 2001; Bakker et al., 2003). These unique characteristics, which are known as job resources and job demands, are believed to greatly influence employees’ professional behaviors, such as work engagement and organizational commitment (Crawford et al., 2010). According to Bakker and Demerouti (2007), when job demands exceed job resources, employees may experience occupational stress and job burnout. They also asserted that experiencing occupational stress and job burnout for a long period of time discourages employees from continuing their profession. Simply said, prolonged stress and burnout lead employees to leave their profession. Extending this idea to the teaching profession, it is logical to argue that when teaching demands surpass teaching resources, teachers will experience stress and burnout that drive them to leave their profession and look for another one.

### Burnout

The notion of burnout was primarily conceptualized by Freudenberger (1974) as “a state of physical, emotional, and mental depletion resulting from work overload, and imbalance between expected and real job reward” (p. 160). This concept was further characterized by Maslach and Leiter (1999) as a state of exhaustion or fatigue that arises as a result of work overload and excessive job demands. Taking these definitions into consideration, Hakanen et al. (2006) described teacher burnout as a constant exhaustion that teachers may experience because of long-term occupational stress. As a multi-faceted variable, burnout comprises three key dimensions, namely “emotional exhaustion,” “depersonalization,” and “reduced personal accomplishment” (Maslach and Jackson, 1981). Emotional exhaustion, according to Maslach and Leiter (2016), pertains to teachers’ sense of emotional emptiness caused by job stressors. As noted by Leiter and Maslach (2016), depersonalization relates to the sense of detachment and indifference that teachers experience in the instructional-learning environments. Finally, as put by Maslach and Leiter (2017), reduced personal accomplishment refers to teachers’ dissatisfaction with their professional performance. In this emotional state, teachers feel that they are not competent enough to positively influence their students’ learning outcomes and lead them toward academic success.

Burned-out teachers, according to Zhang and Zhang (2012), typically demonstrate a higher rate of attrition and turnover intention. They noted that the imbalance that exists between job demands and teachers’ professional capabilities leads them to gradually leave their vocation. In addition, as Jacobson (2016) pointed out, burnout as a debilitative factor noticeably reduces teachers’ productivity at work. The unfavorable consequences of teacher burnout have prompted scholars in the mainstream education and language education domain to explore the internal and external sources of burnout among teachers (e.g., Ju et al., 2015; Ventura et al., 2015; Seifalian and Derakhshan, 2018; Zhaleh et al., 2018; Bodenheimer and Shuster, 2020; Kim and Burić, 2020; Fathi et al., 2021; Xu and Yang, 2021; Bing et al., 2022, to cite a few).

As to the internal sources of teacher burnout, Ju et al. (2015), for instance, assessed the role of emotional intelligence in Chinese teachers’ burnout. For this purpose, 307 school teachers were selected from various schools in china. They were asked to express their ideas regarding the role of emotional intelligence in teacher burnout by answering two reliable questionnaires. The collected answers were analyzed using the structural equation modeling (SEM) approach. Consequently, teachers’ burnout was found to be negatively predicted by their emotional intelligence. Later, in a similar inquiry, Zhaleh et al. (2018) studied this subject in the context of Iran. To do so, two valid scales, namely “Language Teachers’ Conceptions of Intelligence Scale” and “Maslach Burnout Inventory (MBI),” were handed out to 202 English language teachers. The data analysis demonstrated that intelligence was a significant, negative predictor of teacher burnout. In another research, Kim and Burić (2020) inspected the function of teachers’ self-efficacy in their level of burnout. In doing so, 3,002 teachers from Croatia’s primary, secondary, and middle schools were recruited. Then, all participants were invited to fill out two reliable questionnaires measuring self-efficacy and burnout. Consequently, teachers’ self-efficacy had a significant role in predicting their burnout. Besides, to identify the external sources of teacher burnout, Xu and Yang (2021), for example, evaluated the effect of organizational support on Chinese teachers’ burnout. To accomplish this, 351 teachers were selected from primary and secondary schools at random. Following that, two reliable inventories were employed to obtain the needed information. The examination of obtained data indicated that organizational support had a negative impact on teachers’ burnout.

### Job-related stress

The notion of job-related stress has been generally described as a sense of tension threatening one’s wellbeing at work (Lazarus and Folkman, 1984). In the teaching career, this notion has been characterized as “the experience of unpleasant, negative emotions, such as anger, anxiety, tension, frustration, and depression, resulting from some aspects of their work as a teacher” (Kyriacou, 2001, p. 28). As noted by Klassen and Chiu (2011), job-related stress typically emerges when teachers feel that teaching demands and strains do not correspond with their professional knowledge and skills. Teachers with high levels of job-related stress often demonstrate lower job satisfaction, less organizational commitment, and limited work engagement (Klassen and Chiu, 2010; Li et al., 2017; Zhang et al., 2019). Given this, identifying the variables that bring about job-related stress in teachers appears critical. To answer this necessity, many scholars have inquired into the role of emotional and environmental factors in teachers’ job-related stress (e.g., Ghanizadeh and Jalal, 2017; Gonzalez et al., 2017; Troesch and Bauer, 2017; Fathi and Derakhshan, 2019; Alqarni, 2021, among others).

Ghanizadeh and Jalal (2017), for instance, probed the role of job satisfaction in predicting job-related stress. For this aim, 134 English teachers working at different universities in Iran were asked to cooperate in this research by filling out two researcher-made scales. Then, using SEM analysis, the predictive role of job satisfaction was evaluated. As a result, job satisfaction was discovered to be the negative predictor of teachers’ job-related stress. By the same token, Troesch and Bauer (2017) examined the function of teachers’ self-efficacy in their job stress. To this end, 400 teachers answered two questionnaires designed to measure teachers’ self-efficacy and job stress. The gathered answers were then analyzed through hierarchical regression analysis. The analysis outcomes uncovered that self-efficacy beliefs negatively predicted teachers’ stress at work. Likewise, Fathi and Derakhshan (2019) studied the power of teacher self-efficacy in predicting teaching stress. To do so, the “Teachers’ Sense of Efficacy Scale” and the “Teacher Stress Inventory (TSI)” were given to 256 Iranian teachers. Teachers’ answers to the aforementioned scales indicated that teaching stress was negatively predicted by teachers’ self-efficacy.

### Turnover intention

The concept of turnover intention, in a general sense, pertains to “one’s conscious will to look for a job outside the current organization” (Tett and Meyer, 1993, p. 259). Teachers’ turnover intention, in particular, refers to teachers’ inclination to quit the teaching career and look for other working opportunities (Harris and Adams, 2007; Liu and Onwuegbuzie, 2012). As Watlington et al. (2010) mentioned, teachers’ withdrawal from the teaching profession may entail some considerable costs for the educational institution they are working for, such as replacement and training costs. Besides these financial costs, an individual teacher’s departure from the teaching career may have some devastating effects on his/her students’ achievements (Sorensen and Ladd, 2020). The undesirable impacts of teacher turnover intention have led researchers to study the predictors and determinants of this construct (Arnup and Bowles, 2016; De Neve and Devos, 2017; Wang and Hu, 2017; Nazari and Alizadeh, 2021, to cite a few).

To date, Arnup and Bowles (2016), for example, inquired into the role of resilience in predicting Australian teacher’ intention to leave. For this purpose, 160 teachers teaching at different schools in Australia were invited to engage in this study. Close-ended questionnaires were used to collect the required information. The results demonstrated that teachers’ resilience was related to their leaving intention. In addition, resilience was discovered to be a strong determinant of teachers’ intention to leave. As another example, De Neve and Devos (2017) delved into the role of psychological states in teachers’ turnover rates. To do so, the valid measures of autonomy, self-efficacy, and affective commitment were distributed among 272 school teachers. The outcomes indicated that the psychological variables, including autonomy, self-efficacy, and affective commitment, dramatically reduced teachers’ turnover intention. Additionally, in a recent inquiry, Nazari and Alizadeh (2021) examined whether teachers’ turnover intention is the function of teaching experience. To accomplish this, researchers administered two related questionnaires to 325 novice and experienced teachers. Consequently, experienced teachers displayed lower levels of turnover intention than their novice colleagues.

### Previous inquiries into the role of burnout and job-related stress in teachers’ turnover intention

To date, some scholars have inquired into the function of burnout and job-related stress in teachers’ turnover intention (Chika et al., 2016; Xu et al., 2017; Jang and Kim, 2019; Lee, 2019; Mingming et al., 2021; Lai et al., 2022). For instance, Chika et al. (2016) studied job-related stress to examine its potential in predicting teachers’ turnover intention. To meet this aim, 270 Nigerian teachers were invited to complete two scales. The results of multiple regression analysis unraveled the significant role of job-related stress in increasing teachers’ turnover intention. In a similar vein, Xu et al. (2017) explored the impact of occupational stress on teachers’ turnover intention. A set of close-ended questionnaires was utilized to examine participants’ (*N* = 326 school teachers) attitudes regarding the probable effects of occupational stress on teachers’ desire to leave the profession. The results showed that occupational stress directly influenced teachers’ turnover intention.

Furthermore, in his study, Lee (2019) assessed the potential power of burnout in enhancing teachers’ turnover intention. To this end, the electronic versions of the “Turnover Intention Scale (TIS)” and the “Teacher Burnout Questionnaire” were shared with 613 teachers. The findings demonstrated that burnout made a meaningful change in teachers’ turnover rates. Similarly, Mingming et al. (2021) scrutinized the consequences of job burnout for teachers’ turnover intention. In doing so, 992 teachers were randomly selected to respond to the “Job Burnout Scale” and the “Turnover Intention Scale.” The examination of responses exhibited the important role of job burnout in increasing teachers’ turnover intention.

Despite these scholarly endeavors, the research on the role of burnout and job-related stress in teachers’ turnover intention is still in its infancy. Simply said, the function of these emotional variables in teachers’ turnover intention has remained somehow elusive. Moreover, as existing literature demonstrates, no empirical research has probed the predictive role of burnout and job-related stress in distance education contexts. Against this backdrop, the current study aimed to uncover the role of burnout and job-related stress in Chinese English teachers’ turnover intention during distance education.

## Materials and methods

### Participants

Adopting the convenience sampling approach, a sample of 221 English teachers currently working at different colleges and institutes in China was recruited. The rationale behind using the convenience sampling method is that in this sampling procedure “members of the target population are readily selected based on some practical criteria, such as geographical proximity, availability at a certain time, or easy accessibility” (Dörnyei and Csizér, 2012, p. 81). It is worth noting that convenience samples are seldom entirely “convenience-based” but rather somewhat purposeful, which implies that, in addition to the availability and ease of accessibility, participants must possess specific qualities that are tied to the objectives of the inquiry (Mackey and Gass, 2005; Dörnyei, 2007).

The sample of participants comprised both female (*N* = 176) and male (*N* = 45) teachers with various ages (Mean = 40.58, SD = 4.65), ranging from 26 to 58 years old. The teaching experience of participants also varied from 5 to 25 years. The academic degrees of the participants also varied, with 131 teachers holding MA degrees and 90 teachers holding Ph.D. degrees. As to the academic major, participants graduated in three different branches of English, namely linguistics (52%), applied linguistics (33%), and translation (15%). Finally, it should be mentioned that participation in this inquiry was entirely voluntary, and all participants willingly cooperated with scholars in achieving the research purposes.

### Instruments

#### Maslach burnout inventory-educators survey

To measure Chinese English teachers’ burnout, the “Maslach burnout inventory-educators survey (MBI-ES)” was employed. The MBI-ES, designed by Maslach et al. (1996), is a 22-item scale comprising three distinguishing components, namely “reduced personal accomplishment,” “depersonalization,” and “emotional exhaustion.” Participants need to rate these 22 items on a 7-point Likert scale, varying from 0 (never) to 6 (everyday). Sample items involve “I feel used up at the end of the workday” (item 2), “I have become more callous toward people since I took this job” (item 10), “Working with people directly puts too much stress on me” (item 16). A Cronbach alpha reliability coefficient of 0.93 was reported for the MBI-ES in this research.

#### Teacher stress inventory

In order to assess the participants’ stress at work, the “TSI,” validated by Boyle et al. (1995), was utilized. This inventory constitutes 20 items; each was scored on a five-point Likert scale (from 1 = No stress to 5 = Extreme stress). All items commenced with the following question: “As a teacher, how great a source of stress are these factors to you?” The following are three instances of TSI’s items: “*Lack of recognition for good teaching*” (item 3), “*Pupil poor attitude to work*” (item 7), and “*Shortage of equipment and poor facilities*” (item 6). The reliability of this inventory was discovered to be 0.87 in the current study.

#### Turnover intention scale

The “TIS,” developed by Becker and Billings (1993), was used to evaluate Chinese English teachers’ willingness to turnover. The TIS uses a 7-point Likert-type scale, the answers to its items may range from 1 “very strongly disagree” to 7 “very strongly agree.” This scale encompasses four items, as follows: “It is likely I will actively look for a new job in the next year” (item 1), “I often think about quitting teaching” (item 2), “It would take very little change in my present circumstances to cause me to leave teaching” (item 3), and “There’s not too much to be gained by sticking with teaching indefinitely” (item 4). In the current research, a reliability index of 0.91 was found for this scale.

### Procedure

The process of data collection commenced by administering consent forms to 221 Chinese English teachers. Then, it continued with the distribution of three aforementioned scales (MBI-ES, TSI, and TIS) among the participants. This distribution was virtually performed through the Wenjuanxing platform. Upon distribution of the scales, some instructions regarding the completion of the scales were offered to the respondents. The needed information was entirely collected within 3 weeks.

Having gathered the needed information, they were preprocessed to detect the missing and problematic answers. As a result, no missing or questionable answer was discovered. Then, through Kolmogorov–Smirnov and Shapiro–Wilk tests, the normality of answers was evaluated. Following that, the association between burnout, job-related stress, and turnover intention was calculated using Pearson correlation test. Finally, to inspect the role of burnout and job-related stress in predicting Chinese teachers’ turnover intention, the SEM analysis was conducted through AMOS software (version 24).

## Results

To begin, descriptive statistics comprised of central indicators and dispersion of the variables were calculated to offer fundamental information about variables under investigation. The results are evinced in Table 1.

**Table 1:** Central indicators and dispersion of research variables.

		Turnover intention	Burnout	Job-related stress
*N*	Valid	221	221	221
	Missing	0	0	0
Mean		9.73	66.82	52.26
Std. deviation		6.092	20.815	16.131
Skewness		0.968	−0.195	−0.125
Std. error of skewness		0.164	0.164	0.164
Kurtosis		0.021	−0.691	−0.235
Std. error of kurtosis		0.326	0.326	0.326
Minimum		4	22	20
Maximum		28	114	95

As depicted in Table 1, the mean of burnout, job-related stress, and turnover intention turned out to be 66.82, 52.26, and 9.73, respectively. Their deviations were also calculated as 20.815, 16.131, and 6.092, respectively. Moreover, the Skewness and Kurtosis indices were found to be in the range of −2 to 2, implying that the distribution of variables was almost expected. Following the calculation of descriptive statistics, the normality of the collected data was tested using the Shapiro–Wilk and Kolmogorov–Smirnov tests. The outcomes of the tests are displayed hereunder (Table 2).

**Table 2:** The outcomes of Shapiro–Wilk and Kolmogorov–Smirnov tests.

	K-S			S-W		
	Statistic	df	Sig.	Statistic	df	Sig.
Turnover intention	0.174	221	0.000	0.860	221	0.000
Burnout	0.059	221	0.056	0.983	221	0.011
Job-related stress	0.069	221	0.013	0.982	221	0.006

As Table 2 indicated, the normality of the data was violated. Yet, it seems acceptable for a sample with more than 200 participants. Having assessed the normality of gathered data, the probable correlation between burnout, job-related stress, and turnover intention was measured through the Pearson correlation test, the results of which are presented in Table 3.

**Table 3:** The correlation between burnout, job-related stress, and turnover intention.

		Turnover intention	Burnout	Job-related stress
Turnover intention	Pearson correlation	1	0.601^**^	0.522^**^
	Sig. (2-tailed)		0.000	0.000
Burnout	Pearson correlation	0.601^**^	1	0.499^**^
	Sig. (2-tailed)	0.000		0.000
Job-related stress	Pearson correlation	0.522^**^	0.499^**^	1
	Sig. (2-tailed)	0.000	0.000	
	*N*	221	221	221

** Association is significant.

The Pearson correlation test revealed a positive, strong association between burnout and turnover intention (*r* = 0.601, *p* < 0.01). It also uncovered a direct relationship between job-related stress and turnover intention (*r* = 0.522, *p* < 0.01). Furthermore, a close link was discovered between burnout and job-related stress (*r* = 0.499, *p* < 0.01). Following that, through multiple regression method, the role of burnout and job-related stress in predicting teachers’ turnover intention was evaluated. Consequently, both burnout and job-related stress made significant contributions to teachers’ intention to turnover (Table 4).

**Table 4:** Estimates of regression weights for the variables.

			Weight	S.E.	C.R.	*p*
Turnover intention	←	Job-related stress	0.158	0.106	1.498	0.000
Turnover intention	←	Burnout	0.898	0.145	6.186	0.000

Afterward, to identify which variable contributed more significantly to teachers’ turnover intention, standardized regression weights were calculated. As a result, burnout was found to be the stronger predictor of teachers’ turnover intention in that it uniquely explained 69 percent of the variance in the teachers’ turnover rates (Table 5).

**Table 5:** Standardized regression weights for the variables.

			Estimate
Turnover intention	←	Job-related stress	0.11
Turnover intention	←	Burnout	0.69

**Figure 1:**
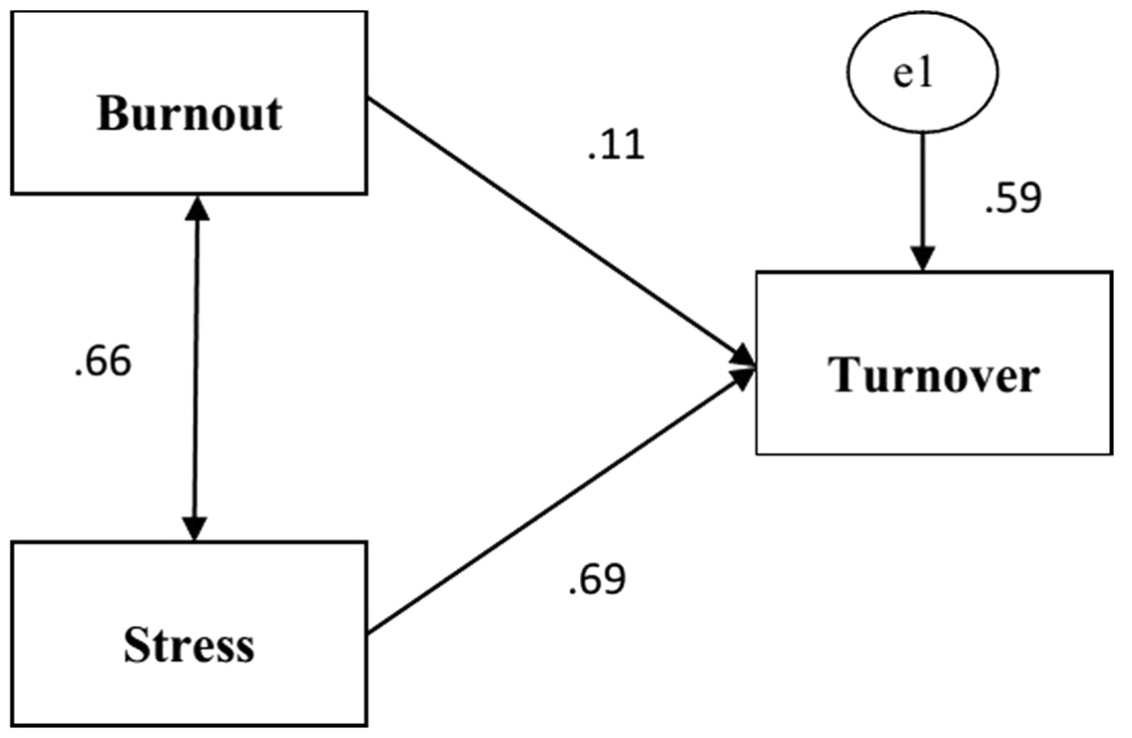
Prediction model with standardized values.

The role of burnout and job-related stress in predicting teachers’ turnover intention was portrayed using SEM analysis. The prediction model with standardized estimates is provided below (Figure 1).

## Discussion

The objectives of the current inquiry were, in the first place, to test the associations between burnout, job-related stress, and turnover intention; and in the second place, to probe the role of burnout and job-related stress in predicting Chinese English teachers’ turnover intention. As to the first objective of this study, the results of the Pearson test demonstrated that both burnout and job-related stress were closely related to turnover intention. The outcomes of the correlation test uncovered a similar connection between job-related stress and burnout as well. Concerning the second objective, the SEM analysis outcomes revealed that burnout and job-related stress served an undeniable role in increasing Chinese English teachers’ turnover intention.

The result of this research concerning the strong link between burnout and turnover intention is in line with those of Lee (2019), who observed a strong link between teachers’ burnout and their turnover intention. It also supports the findings of Mingming et al. (2021), who discovered a direct association between burnout and teachers’ intention to leave the teaching profession. Moreover, the outcome of the present inquiry about the significant association between job-related stress and turnover intention backs up Chika et al.’s (2016) findings, which demonstrated that teachers’ job-related stress is intertwined with their turnover intention. It also resonates with the outcomes of Xu et al. (2017), who reported that teachers’ turnover intentions are tied to the prolonged stress they feel at work. Additionally, the present study’s outcome regarding the close relationship between burnout and job-related stress is congruent with Xu and Yang’s (2021) results, which showed that burnout is tightly associated with job stress.

Besides, the results of this investigation on the power of burnout and job-related stress in predicting teachers’ turnover intention can be logically justified in light of Bakker and Demerouti’s (2007) JD-R model. According to this conceptual model, those employees who experience stress and burnout for a long period of time are more prone to the turnover phenomenon. That is, employees with a high amount of job stress and burnout tend to quit their careers and search for another profession. It is mainly due to the fact that prolonged occupational stress and job burnout substantially reduce employees’ motivation, interest, and willingness to continue their current profession (Bakker and Demerouti, 2007). Considering this theoretical model, burned-out and anxious teachers are less inclined to pursue the teaching vocation. It indicates that the higher the teachers’ occupational stress and job burnout, the stronger their turnover intention. This result confirms the idea of Lu and Gursoy (2016), who asserted that burnout substantially enhances teachers’ intention to turnover. This outcome also supports Skaalvik and Skaalvik’s (2016) assertion regarding the predictive role of occupational stress. They claimed that being overwhelmed with occupational stress remarkably decreases teachers’ intention to work.

## Conclusion, implications, and limitations

This research was undertaken to unravel the association between job-related stress, burnout, and turnover intention and the role of job-related stress and burnout in predicting Chinese English teachers’ turnover intention during distance education. The results obtained from correlational and SEM analysis evinced the high potential of job-related stress and burnout in predicting Chinese English teachers’ turnover intention. It indicates that in the absence of job-related stress and burnout, teachers are less likely to quit the teaching career. Simply said, job-related stress and burnout can result in enhanced turnover intention among teachers.

The outcomes of this inquiry might have some valuable implications for language teachers in any educational environment, notably distance education contexts. Given the high potential of job-related stress in predicting teachers’ turnover intention, practicing teachers are required to constantly engage in teacher training courses to learn how to deal with job-related stress. For the same reason, they also need to attend professional development programs like workshops and conferences to update their knowledge of coping strategies. The results of the present investigation also appear to be instructive for teacher trainers. Owing to the significant role of job-related stress in increasing teachers’ turnover intention, teacher educators are highly expected to teach practicing teachers how to cope with stressful situations at work. Upon equipping teachers with appropriate coping strategies, teacher educators can substantially decrease the rate of turnover among in-service teachers. Additionally, the findings of this research may entail some informative and fruitful implications for educational managers. Considering the direct effects of burnout and job-related stress on teachers’ turnover intention, educational managers must provide teachers with a comfortable, stress-free working environment. Simply said, they need to reduce the professional demands and pressures that are made upon teachers in educational contexts. As a result, they can dramatically decrease the amount of turnover among practicing teachers.

It is worth mentioning that the outcomes of the present research should be interpreted in light of three significant limitations. First, the current investigation was entirely performed in China, which is an “English as a Foreign Language (EFL)” country. Hence, the results of this investigation are only transferable to EFL contexts. The educational researchers are thus advised to explore the role of job-related stress and burnout in teachers’ turnover intention in an “English as a Second Language” country to find any meaningful changes in the outcomes. Second, the mediating or moderating effects of situational variables like teaching experience on teachers’ turnover intention were disregarded in this research. It would be inspiring to measure the effects of these variables in future studies. Third, only close-ended questionnaires were used to elicit participants’ viewpoints regarding the interrelationships of the variables under investigation. Other data collection instruments like interviews and open-ended questionnaires would help future studies to gather more comprehensive information.

## Data availability statement

The original contributions presented in the study are included in the article/Supplementary material, further inquiries can be directed to the corresponding author.

## Ethics statement

The studies involving human participants were reviewed and approved by Zhoukou Normal University Academic Ethics Committee. The patients/participants provided their written informed consent to participate in this study.

## Author contributions

The author confirms being the sole contributor of this work and has approved it for publication.

## Funding

This study was supported by Henan Province Philosophy and Social Science Project—Collection and Arrangement of Fuxi Culture Documents in the District of Huaiyang (NO.: 2022BYY030).

## Conflict of interest

The author declares that the research was conducted in the absence of any commercial or financial relationships that could be construed as a potential conflict of interest.

## Publisher’s note

All claims expressed in this article are solely those of the authors and do not necessarily represent those of their affiliated organizations, or those of the publisher, the editors and the reviewers. Any product that may be evaluated in this article, or claim that may be made by its manufacturer, is not guaranteed or endorsed by the publisher.
